# Promotion of Cobalt Oxide Catalysts by Acid-Etching and Ruthenium Incorporation for Chlorinated VOC Oxidation

**DOI:** 10.1021/acs.iecr.3c04045

**Published:** 2024-02-13

**Authors:** Amaya Gil-Barbarin, José Ignacio Gutiérrez-Ortiz, Rubén López-Fonseca, Beatriz de Rivas

**Affiliations:** Chemical Technologies for Environmental Sustainability Group, Department of Chemical Engineering, Faculty of Science and Technology, University of the Basque Country UPV/EHU, Barrio Sarriena s/n, Leioa E-48940, Bizkaia, Spain

## Abstract

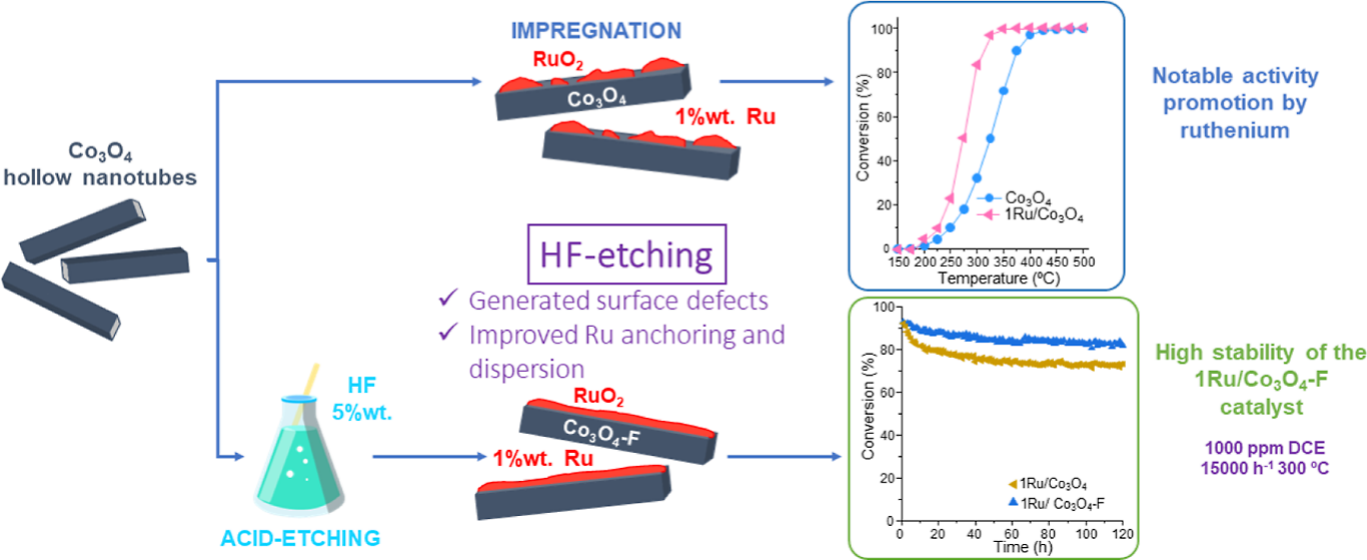

In this work, Ru-promoted cobalt oxide catalysts with a nanotube morphology were prepared by a synthesis route based on the Kirkendall effect followed by an acid treatment and subsequent optimized Ru impregnation. The resulting samples were thoroughly characterized by means of N_2_ physisorption, X-ray energy-dispersive spectroscopy, X-ray diffraction, scanning electron microscopy techniques, X-ray photoelectron spectroscopy, and temperature-programmed techniques (O_2_-temperature-programmed desorption, H_2_-temperature-programmed reduction, and temperature-programmed oxidation) and evaluated in the gas-phase oxidation of 1,2-dichloroethane. It has been demonstrated that Ru addition improves the oxygen mobility as well as the amount of Co^2+^ and O_ads_ species at the surface by the formation of the Ru–O–Co bond, which in turn governs the performance of the catalysts in the oxidation reaction. Moreover, the acid-etching favors the dispersion of the Ru species on the surface of the catalysts and strengthens the interaction among the noble metal and the cobalt oxide, thereby improving the thermal stability of the Ru-promoted oxides. Thus, the resulting catalysts are not only active, as the chlorinated pollutant is efficiently converted into deep oxidation products at relatively low temperatures, but also quite stable when operating for 120 h.

## 1 Introduction

Emissions of chlorinated volatile organic compounds (Cl-VOCs) have increased in recent years due to the development of modern industry. Along with the environmental impacts (increase in photochemical smog, generation of ground-level ozone, and depletion of stratospheric ozone), the negative effects on human health caused by these emissions have led to the search for technologies for the control and removal of these compounds.^1,2^ Among the available processes (adsorption, membrane separation, thermal incineration, and biodegradation), catalytic oxidation is presented as an optimal strategy, owing to its high efficiency in the elimination of trace amounts of Cl-VOCs at relatively low temperatures.^3^

In this sense, the choice of a suitable catalyst (in terms of activity, selectivity toward deep oxidation products, and stability) is essential. Bulk transition-metal oxides, especially Mn, Cu, Fe, or Co oxides, have been extensively applied in Cl-VOC oxidation as a result of their notable catalytic activity and low cost compared with noble metal catalysts.^4−6^ Particularly, cobalt-based catalysts have exhibited excellent results in several catalytic processes related to environmental protection.^6−9^ The high catalytic activity of this oxide is attributed to its high bulk oxygen mobility, surface area, abundant defects, and the easy formation of highly active oxygen vacancies.^10,11^ However, these oxides present some drawbacks such as its slow oxidation–reduction and a high chlorination activity.^12^ To overcome these problems, incorporation of controlled amounts of promoters may be applied.^10,12^ Noble metals are considered as a good alternative since the amount to be used as a promoter is eventually small, which would not make the catalyst much more expensive. Nonetheless, some of the most commonly used noble metals (Pt and Pd) are very sensitive to Cl poisoning which greatly decreases their stability in the oxidation of chlorinated compounds. On the contrary, ruthenium has been widely applied in the gas-phase oxidation of HCl to Cl_2_ owing to the remarkable dechlorination efficiency of Ru species.^13^ Thus, the Cl species generated from the cleavage of the C–Cl bonds during the oxidation of the chlorinated hydrocarbon are favorably removed from the catalyst surface as Cl_2_ owing to the activity of Ru in the Deacon reaction (2HCl + 1/2O_2_ → Cl_2_ + H_2_O). Moreover, Ru is relatively inexpensive (2–3 times cheaper than Pt and 5–6 times less expensive than Pd), and it has been shown to be quite active in the catalytic oxidation of light hydrocarbons such as propane.^14^ However, RuO_*x*_ is known to be thermally unstable, leading to volatilization of Ru species in catalytic oxidation processes at high temperatures and resulting in catalysts with poor stability when operating for a long time.^15^

In this sense, the beneficial role of the promoter in terms of enhanced stability relies on its dispersion and interaction with the active phase. Several strategies can be followed to modulate promoter-active phase interactions: tune the characteristics of the bulk oxide acting as a support for the promoter (by altering its morphology and/or chemical composition), change the features of noble metal (by controlling its particle size), and modify the overall structure of the promoted oxide catalyst (by thermal treatment with controlled atmospheres).^16^ It has been demonstrated that structural defects on the support surface boost metal–support interaction. Zhang et al. showed that oxygen vacancies in TiO_2_ led to strong metal–support interaction between Cu and the support and enhanced the hydrogenation reaction of CO_2_ to methanol.^17^ Similarly, Wan et al. found that surface oxygen vacancies were appropriate sites to anchor single atomic metal sites.^18^ As well as oxygen vacancies, steps on the support have higher adsorption energy and enhanced the interaction between metal particles and support compared with terraces or smooth surfaces, as revealed by Gong et al.^19^ In conclusion, the introduction of defects in the support could improve metal–support interactions by enhancing the covalent bonds between the active metal and the support.^15^ There are numerous methods available for this purpose, among which acid-etching is a widely employed technique to modulate surface composition and create structural defects on metal oxides.^20,21^

Thus, in this work, Co_3_O_4_ and Ru-promoted Co_3_O_4_ catalysts were synthesized and examined for the gas phase removal of 1000 ppm of 1,2-dichloroethane (DCE) as a model-chlorinated VOC. The cobalt oxide support was etched with an aqueous HF solution (5 wt %) to generate surface defects for ruthenium oxide stable anchoring. Subsequently, Ru was deposited on the etched support (0.5, 0.75, and 1 wt %) by an impregnation method to enhance the activity of cobalt oxide in the catalytic removal of chlorinated VOCs. The as-synthesized oxides were extensively characterized by several techniques including N_2_ physisorption, energy-dispersive X-ray spectroscopy (EDX), X-ray diffraction (XRD), Raman spectroscopy, scanning electron microscopy (SEM), high-angle annular dark field scanning transmission electronic microscopy (STEM-HAADF), temperature-programmed reduction with hydrogen (H_2_-TPR), temperature-programmed desorption of oxygen (O2-TPD), temperature-programmed oxidation (TPO) with oxygen, and X-ray photoelectron spectroscopy (XPS).

## 2 Experimental Methods

### 2.1 Synthesis of the *x*Ru/Co_3_O_4_–F Catalysts

The preparation of the catalysts involved three consecutive steps, namely, the synthesis of the parent bulk cobalt oxide, its modification by acid-etching and finally the incorporation of the noble metal (Ru).

#### 2.1.1 Preparation of the Co_3_O_4_ Samples

For the preparation of the Co_3_O_4_ nanotubes by the Kirkendall effect, a 0.03 M ethanolic solution of cobalt acetate tetrahydrate (Alfa Aesar) was prepared, to which a 0.17 M ethanolic solution of urea (Fluka) was added under stirring. This mixture was kept under stirring for 4 h at 65 °C using a jacketed reactor with reflux to maintain the temperature. Next, the suspension was cooled and then filtered (0.22 μm) to obtain the cobalt hydroxyacetate precursor. The solid was washed with ethanol three times and then dried in an oven at 80 °C for at least 12 h. Finally, the precursor was calcined using a rotary furnace with a flow of 500 cm^3^ min^–1^ of a 5%O_2_/He stream. The temperature was first increased to 350 °C using a heating ramp of 1 °C min^–1^ and then kept for 3 h. During the heating process, the oxidation of the C, H, and Co atoms of the Co acetate hydroxide occurred first on the surface of the precursor. In this way, a film of Co_3_O_4_ was formed on the surface and the newly exposed C, H, and Co atoms emigrated to the surface to react with the oxygen. This process was repeated with the continuous outflow to the surface of the cobalt acetate hydroxide, resulting in the formation of cavities and the subsequent generation of the hollow Co_3_O_4_ nanotubes. At this temperature (350 °C), the transformation into Co_3_O_4_ was expected to be fully achieved. However, the oxide was subjected to a new calcination process at 500 °C to produce a thermally stabilized catalyst under the selected reaction conditions for DCE oxidation. This second calcination step was carried out using a heating rate of 1 °C min^–1^ followed by an isothermal event for 3 h (500 °C).^22^

#### 2.1.2 Acid Modification of the Co_3_O_4_

Once the Co_3_O_4_ nanotubes were obtained, the next step was the acid treatment of these nanotubes. For this purpose, 1 g of the oxide was taken and placed in a beaker to which 2 mL of 1.25 M HF solution was added. The beaker was then placed in an ultrasonic bath for 30 min at room temperature. The content of the beaker was subsequently filtered to recover the modified Co_3_O_4_ and washed with distilled water several times to remove any remaining acid solution. Finally, it was dried in an oven at 110 °C overnight. Finally, it was calcined in a muffle furnace under static air conditions at 500 °C using a heating ramp of 1 °C min^–1^. This temperature was maintained for 3 h. The resulting sample was named as Co_3_O_4_–F.

#### 2.1.3 Incorporation of Ruthenium

The last step to produce the fully formulated catalysts was the incorporation of ruthenium by means of an optimized impregnation method. Hence, an adjusted amount of RuCl_3_ (Alfa Aesar) was weighed to achieve the desired Ru content (the Ru/Co_3_O_4_ mass ratios were 0.5:100, 0.75:100, and 1:100) and then dissolved in distilled water to obtain a 0.2 M solution. Then, a certain volume (10, 15, or 20 mL) of 15% H_2_O_2_ (Sigma-Aldrich) was added dropwise under stirring, using a peristaltic pump with a flow rate of 5 cm^3^ min^–1^. This solution was then transferred to a water bath where the temperature was raised up to 95 °C. The system was kept under stirring at this temperature for 2 h (with a reflux system to avoid evaporation of water) to obtain the RuO_2_ suspension. After this time interval, the suspension was cooled, and the Co_3_O_4_ powder was added to the mixture. Subsequently, the mixture was heated to 50 °C and kept under stirring at this temperature for 24 h.^23^ Afterward, the samples were dried in a rotary evaporator with a rotation speed of 20 rpm at 40–50 °C. Finally, they were calcined in a muffle furnace under static air conditions at 500 °C using a heating ramp of 1 °C min^–1^ and maintained under isothermal conditions for 3 h. The as-synthesized catalysts were denoted as *x*Ru/Co_3_O_4_–F, where x is the Ru loading of the sample. For comparative purposes, a reference catalyst was also prepared (1Ru/Co_3_O_4_). In this case, the cobalt oxide nanotubes were not previously treated with HF.

### 2.2 Characterization Techniques

Textural properties of the obtained samples were examined by N_2_ physisorption at the temperature of liquid nitrogen (−196 °C). The experiments were carried out in a Micromeritics TRISTAR II 3020 instrument, and the specific surface area and mean pore size were calculated using the Brunauer–Emmett–Teller (BET) and Barrett–Joyner–Halenda methods, respectively. Prior to the analysis, the samples were degassed at 200 °C for 10 h with N_2_ flow. The structural properties of the synthesized catalysts were examined by XRD in powder and Raman spectroscopy. XRD diffraction patterns were taken on an automatic diffractometer model X’Pert PRO from PANalytical using Cu Kα radiation (λ = 1.5406 Å) and a Ni filter at 40 kV and 40 mA. The measurement conditions were optimized to an angular range between 5 and 80° at 2θ with a step size of 0.026° and counting time of 498.3 s per step. International Centre for Diffraction Data (ICDD) database cards were employed for phase identification. Raman spectra were collected on a Renishaw InVia Raman spectrometer using a 514 nm laser source (ion-argon laser, Modu-Laser) and a Leica 50 × Plan lens, scanning from 150 to 1500 cm^–1^. For each spectrum, 20 s were employed, and 10 scans were accumulated with 10% of the maximum power in the spectral window (2 mW).

The external morphology of the synthesized oxides was studied by means of SEM. For each measurement, a small amount of powdered sample was taken and deposited on a carbon strip in a sample holder. SEM images were obtained on a Hitachi S-4800 scanning electron microscope with cold cathode field emission gun at a working voltage of 10 kV and 5 A. The microscope was equipped with a high-resolution CCD camera. Scanning transmission electron microscopy-high angle annular dark field (STEM-HAADF) images with elemental maps were obtained on a ThermoFisher Scientific/FEI Titan electron microscope operating at 300 kV and equipped with a CESCOR Cs CEOS corrector and Oxford Instruments Ultim Max EDX detector. All these components were operated with a Fischione HAADF detector in the STEM mode. Before the measurement, the samples were sonicated in ethanol and dropped on an amorphous carbon film supported on a copper grid. The collected maps were presented in the form of a matrix of colored pixels with the intensity corresponding to the amount of the element.

Redox properties of the catalysts were evaluated by means of H_2_-TPR. The analyses were performed on a Micromeritics Autochem 2920 instrument equipped with a thermal conductivity detector (TCD) detector. Previous to the analysis, the sample was pretreated in a 5%O_2_/He stream at 300 °C for 1 h and then cooled to room temperature. Subsequently, a stream of 50 cm^3^ min^–1^ of 5%H_2_/Ar was flown through the sample from room temperature to 500 °C (10 °C min^–1^) and maintained for 0.5 h. A cold trap was employed to prevent water generated by the reduction step from interfering with the TCD detector. In order to provide more information about the nature of the mobile oxygen species, O_2_-temperature-programmed desorption experiments were also carried out on the same apparatus. Before the analysis, the sample (100 mg) was pretreated in 5%O_2_/He stream at 500 °C for 15 min and then cooled to 50 °C. Next, after being purged with helium for 0.5 h, the catalyst was heated to 900 °C for 0.5 h with a heating ramp of 10 °C min^–1^ while following the signal of the desorbed oxygen (*m*/*z* = 32) with a Hidden HPR 20 EGA mass spectrometer.

The surface chemical composition was evaluated by X-ray photoelectronic spectroscopy (XPS). The measurements were conducted on a Kratos AXIS Supra spectrometer using 120 W Al Kα monochromatic radiation source with a pass energy of 160 eV for the general survey and 20 eV for the specific spectra. The spectra were adjusted using CasaXPS 2.3.16 software, which models Gauss-Lorentzian contributions, after a background subtraction (Shirley). The concentrations were calculated by correcting the values with relative atomic sensitivity factors (Scofield).

X-ray energy-dispersive spectroscopy (EDX) and TPO were employed to evaluate the eventual chlorine accumulation and coke generation over the used catalysts. EDX results were obtained on a JEOL JSM-6400 scanning electron microscope coupled to an INCA EDX X-sight Si Series (Li) pentaFET Oxford detector with window and INCA 350 data acquisition and power processing allowing accurate, online, and mapped analysis. EDX spectra were measured at 20 kV, 1 nA, and at a working distance of 10 mm. The samples were deposited on an amorphous carbon film supported on a copper grid and placed inside the microscope under high vacuum. TPO analyses were conducted in the TGA 550 thermobalance. The thermobalance was coupled to a Pffeifer Vacuum DUO 2.5 mass spectrometer. Previously to the oxidation process, all the samples (100 mg) were pretreated in a 5%O_2_/He flow at 110 °C for 1 h. Subsequently, the temperature was increased to 800 °C with a heating rate of 10 °C min^–1^, and this temperature was held for 0.5 h.

### 2.3 Catalytic Behavior Evaluation

The catalytic activity of the catalysts was determined in a bench-scale fixed bed reactor (MICROACTIVITY-Reference MAPXL1M6 model, supplied by PID Eng&Tech S.L.) fully monitored by a computer. For each experiment, 0.85 g of the catalyst (0.08–0.16 mm) was diluted with quartz (0.5–0.8 mm) up to a total volume of 2 cm^3^. The catalytic bed was introduced into a quartz reactor of 300 mm length and 10 mm internal diameter, in which a K-type thermocouple was inserted for temperature control. The reactor was fed with 1000 ppm of DCE diluted in 500 cm^3^ min^–1^ of dry air. The corresponding weight (WHSV) and gas (GHSV) hourly space velocities were 35,000 mL g^–1^ h^–1^ and 15,000 h^–1^, respectively. The feeding of the gaseous reagents was controlled by mass flow controllers (EL-FLOW from Bronkhorst High-Tech B.V.) while a syringe pump (kdScientific 200) was used to feed the DCE and water (runs under humid conditions). The position of the liquid injection was electrically heated to ensure the complete evaporation of the reactants. To avoid possible fluctuations in the inlet stream, before reaching the reactor, the feed was homogenized in a 2 L mixer. Catalytic activity was measured from 150 to 500 °C in steps of 25 °C, and conversion data were taken at steady state. For the analysis of molecular chlorine and hydrogen chloride, both volumetric and potentiometry methods were employed, respectively.^22^

## 3 Results and Discussion

### 3.1 Physical-Chemical Characterization of Co_3_O_4_ and 1Ru/Co_3_O_4_ Catalysts

Textural properties of the synthesized catalysts were evaluated by N_2_ physisorption. The adsorption/desorption isotherms as well as the pore size distributions are displayed in Figure S1, Supporting Information. The bare Co_3_O_4_ showed a type IV isotherm with type H1 hysteresis loop at a relative pressure of *P*/*P*_0_ = 0.85, typical of mesoporous solids with cylindrical channels.^24^ After the incorporation of ruthenium (presumably in the form of RuO_*x*_), no relevant differences in the type of isotherm or hysteresis cycle were found, suggesting that the mesoporous structure was mostly retained upon Ru impregnation. A bimodal pore size distribution was found, where small pores around 25 Å were due to the inner of the nanoparticles; meanwhile, the larger pores around 300 Å were originated by the agglomeration of the nanoparticles.^25^ Results of BET surface area, pore volume, and mean pore diameter are shown in Table 1. The Ru-modified sample exhibited a slightly lower surface area (16 m^2^ g^–1^) and pore volume (0.04 cm^3^ g^–1^), probably due to the partial blockage of the cobalt oxide (19 m^2^ g^–1^, 0.05 cm^3^ g^–1^) pores. As estimated by EDX, the Ru content was 1 wt %.

**Table 1:** Textural, Structural, and Redox Properties of the Modified Co_3_O_4_ Catalysts^a^

catalyst	Ru content, wt % (EDX)	*S*_BET_, m^2^ g^–1^	*V*_pore_, cm^3^ g^–1^	*d*_pore_, Å	*D*_Co_3_O_4__, nm (XRD)	*D*_Co_3_O_4__, nm (STEM-HAADF)	low-*T* reduction peak, °C	high-*T* reduction peak, °C	O_2_ desorbed at low *T*, μmol g^–1^
Co_3_O_4_		19	0.05	200	22	n.a.	260	330	41
1Ru/Co_3_O_4_	1.0	16	0.04	160	25	n.a.	160	245	63
Co_3_O_4_–F		9	0.02	190	35	n.a.	260	335	40
0.5Ru/Co_3_O_4_–F	0.5	10	0.03	200	37	31	140	225/265	52
0.75Ru/Co_3_O_4_–F	0.6	10	0.03	200	36	32	140	220/260	49
1Ru/Co_3_O_4_–F	0.9	10	0.02	180	36	34	145	230/270	60

a n.a.: not analyzed.

**Figure 1:**
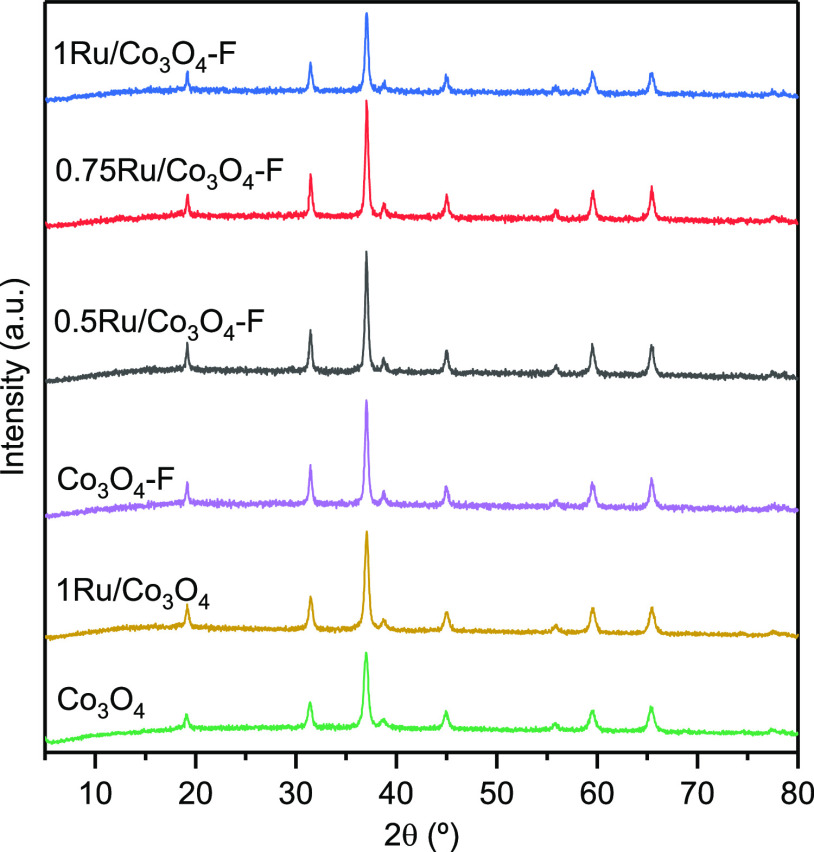
XRD patterns of the modified Co_3_O_4_ samples.

The XRD patterns of the samples are presented in Figure 1. Both bare Co_3_O_4_ and 1Ru/Co_3_O_4_ catalysts exhibited the same diffraction peaks corresponding to the cubic Co_3_O_4_ oxide at 2θ = 19.2, 31.4, 37.0, 38.7, 45.0, 55.8, 59.5, and 65.4°, which were identified with the (111), (220), (311), (222), (400), (422), (511), and (440) planes, respectively (ICDD 00-042-1467). No diffraction peaks related to the CoO phase or other impurities were visible. For the 1Ru/Co_3_O_4_ sample, no diffraction peaks corresponding to ruthenium or ruthenium oxides were detected, possibly due to the low concentration of the metal and its high dispersion on the surface.^11^ By applying the Scherrer equation to the diffraction peak associated with the (311) crystal plane, the average crystallite size was estimated (Table 1). The crystallite size was 22 nm on pure Co_3_O_4_ and slightly larger on 1Ru/Co_3_O_4_ catalyst (25 nm).

**Figure 2:**
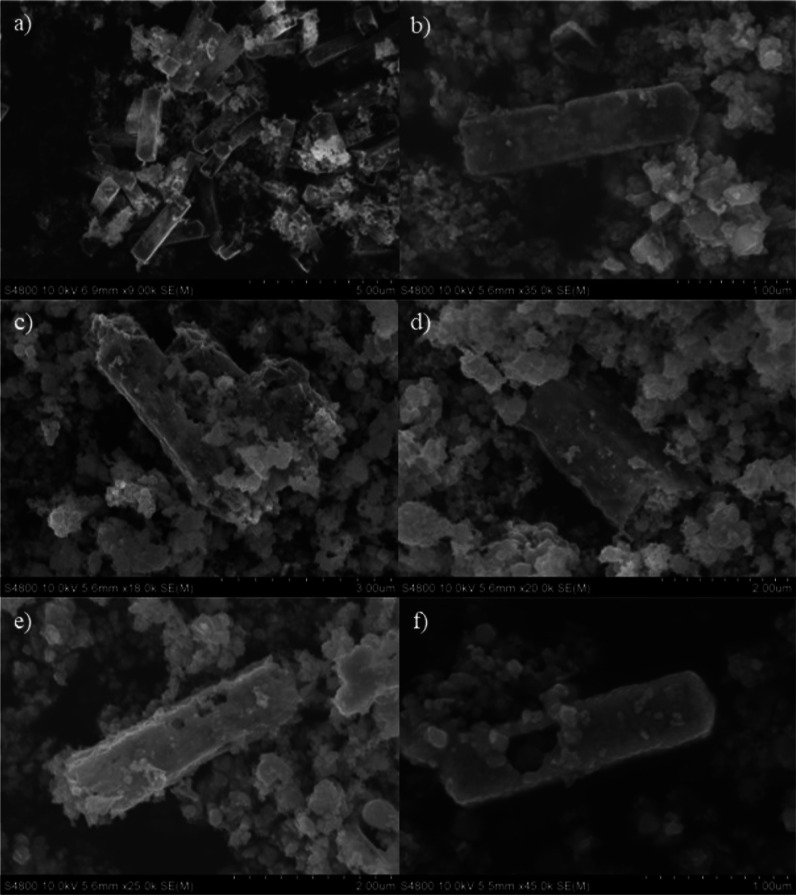
SEM images of the modified Co_3_O_4_ catalysts after calcination at 500 °C. (a) Co_3_O_4_, (b) 1Ru/Co_3_O_4_, (c) Co_3_O_4_–F, (d) 0.5Ru/Co_3_O_4_–F, (e) 0.75Ru/Co_3_O_4_- F, and (f) 1Ru/Co_3_O_4_–F.

The external morphology of the catalysts was examined by SEM (Figure 2). The Co_3_O_4_ sample (Figure 2a) exhibited a reasonably uniform prism morphology. From the images of the broken areas, the desired hollow interior of the tubes could be distinguished. These nanotubes were approximately 2–3 μm in length and 0.5 μm in width. Besides, it is remarkable that the walls of the nanotubes were made of the assembly of nanoparticles of a few nanometers in size. After the incorporation of ruthenium, the regular nanotube morphology was partially lost probably due to sintering during the second calcination process (500 °C). Thus, a substantial number of nanoparticles (derived from the nanotube wall breakage) were found together with the nanotubes (Figure 2b). Nonetheless, no appreciable changes in the morphology were noticed after Ru impregnation.

**Figure 3:**
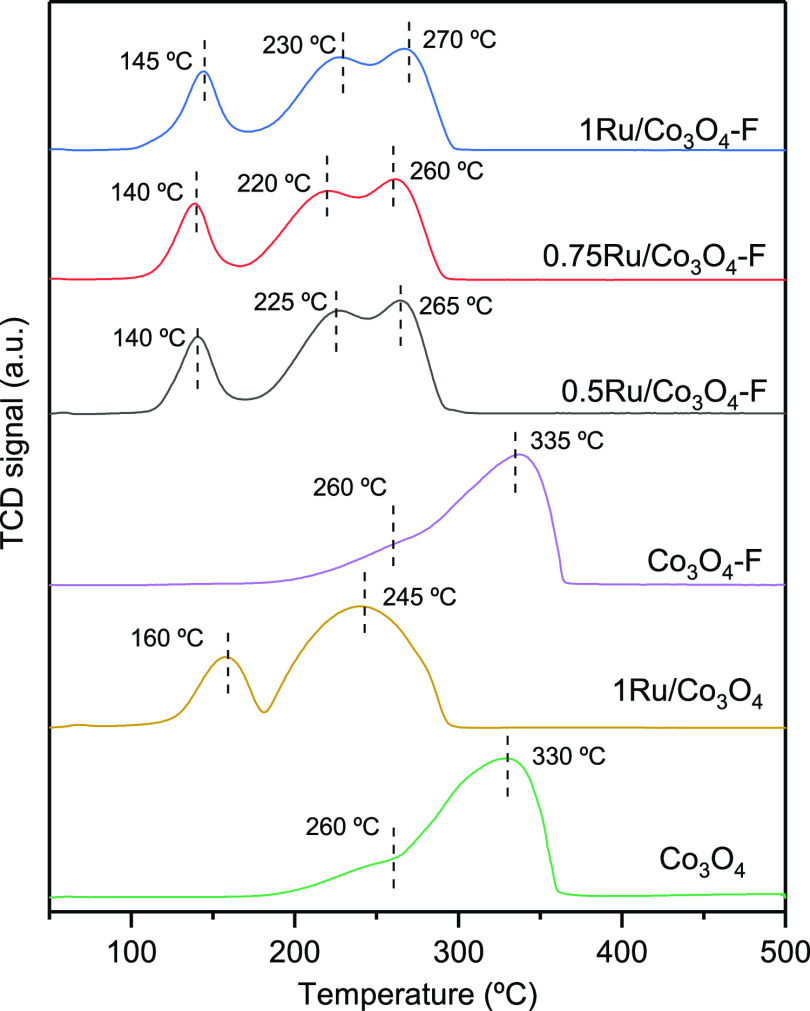
H_2_-TPR profiles of the modified Co_3_O_4_ catalysts.

It is widely accepted that the activity of metal oxide catalysts for oxidation reactions is mostly influenced by the abundance of highly mobile oxygen species. To further investigate the impact of the noble metal deposition on the reducibility of cobalt oxide, H_2_-TPR experiments were conducted. The reduction profiles in the 50–500 °C temperature range of the Co_3_O_4_ and 1Ru/Co_3_O_4_ samples are presented in Figure 3. For the bare Co_3_O_4_ catalyst, two overlapping signals were distinguished between 200 and 360 °C, in agreement with a two-step reduction mechanism (Co^3+^ → Co^2+^ → Co^0^).^26^ After the incorporation of RuO_2_ (as will be shown later by XPS), the reduction process shifted toward substantially lower temperatures. Thus, two distinct reduction peaks at 100–180 and 180–300 °C were observed. Recall that the total reduction of the blank cobalt oxide was attained over 360 °C. It could therefore be concluded that the deposition of Ru onto the spinel structure markedly promoted its reduction at lower temperatures. Some authors agree that the decrease in the reduction temperature of Ru-impregnated samples was due to the hydrogen spillover effect. Ru oxide was reduced at significantly lower temperatures (according to literature, between 100 and 175 °C^13,27^) in comparison with Co_3_O_4_. Then, the metallic ruthenium could act as a reduction nucleus and generate atomic hydrogen that spread to Co_3_O_4_ to accelerate its reduction.^11^ In view of the shape of the reduction profile, it could be deduced that the process took place via the same two-step mechanism. Hence, Co_3_O_4_ was first reduced to CoO at about 160 °C and subsequently fully transformed into metallic Co at 245 °C. It must be pointed out that since the amount of Ru in the sample was very small, the H_2_ uptake attributed to the reduction of ruthenium oxide was expected to overlap with the first reduction peak of Co_3_O_4_.

**Figure 4:**
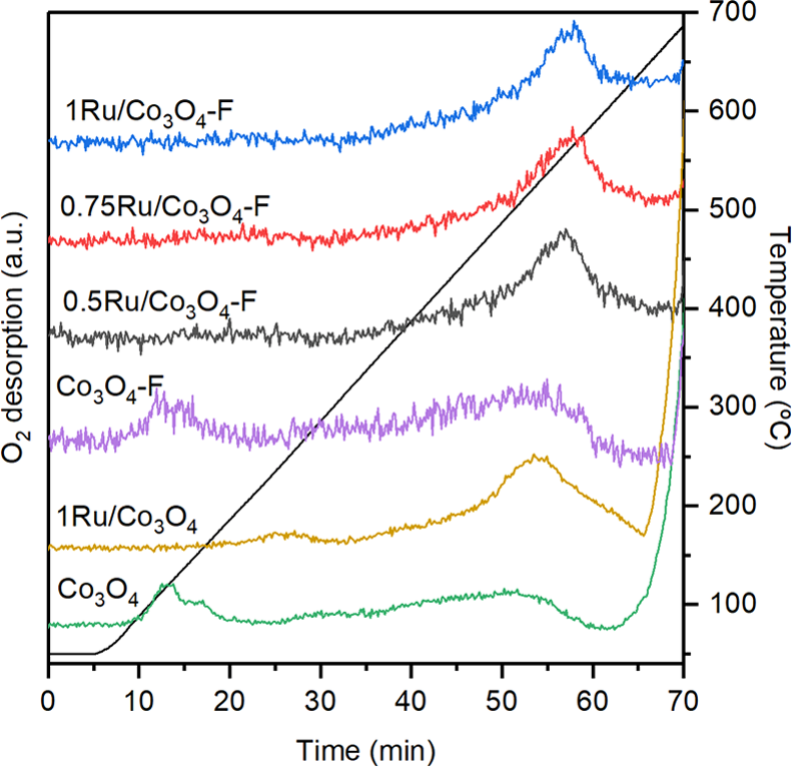
O_2_-TPD profiles of the modified Co_3_O_4_ catalysts.

As a complementary technique to evaluate the mobility of surface and bulk oxygen species, O_2_-TPD measurements were carried out in the 50–900 °C temperature range. Generally, surface oxygen species (both adsorbed and lattice species) are desorbed below 700 °C; meanwhile, bulk oxygen species need temperatures higher than 700 °C to desorb.^28^Figure 4 includes the O_2_-TPD profiles of the bare and Ru-promoted Co_3_O_4_ catalysts. Note that the intense desorption signal above 700 °C that appeared over both samples due to thermal decomposition of Co_3_O_4_ is not shown. As regards surface oxygen species, several desorption peaks ascribed to oxygen species with varying nature could be distinguished. Thus, pure Co_3_O_4_ exhibited two overlapping desorption peaks between 120 and 150 °C, ascribed to surface-adsorbed oxygen species, which were not present in the promoted counterpart. Some authors state that Ru doping diminishes the amount of surface-adsorbed oxygen due to the formation of Ru–O–Co bond or partial blockage of pore structure by Ru species.^29^ At higher temperatures, a weak desorption peak could be noticed at 245 and 290 °C for the 1Ru/Co_3_O_4_ and Co_3_O_4_ samples, respectively. Finally, above 350 °C, a markedly intense desorption peak over the 1Ru/Co_3_O_4_ catalyst could be identified, corresponding to the desorption of surface lattice oxygen species.^30^ From the integration of the desorption profiles, it was found that the amount of desorbed oxygen species up to 700 °C increased from 41 μmol O_2_ g^–1^ over the bare Co_3_O_4_ catalyst to 63 μmol O_2_ g^–1^ over the Ru-containing sample (Table 1). In sum, results of H_2_-TPR and O_2_-TPD revealed a superior oxygen mobility and a higher amount of surface oxygen species in the 1Ru/Co_3_O_4_ catalyst, features of high interest in chlorocarbon combustion.

**Figure 5:**
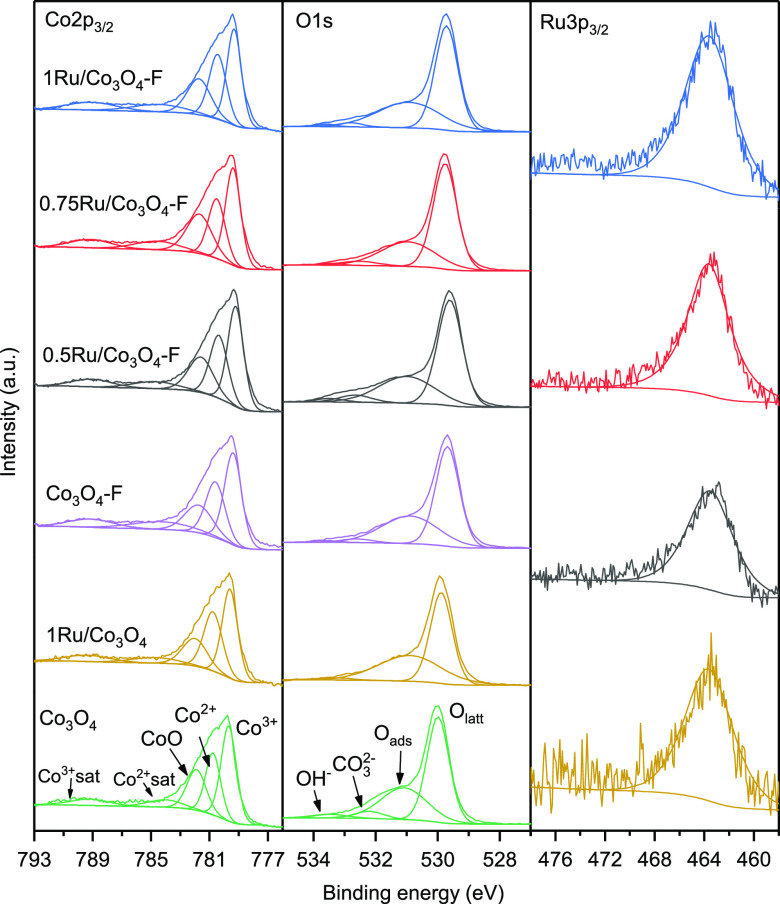
XPS profiles of the modified Co_3_O_4_ catalysts.

XPS analysis was employed to further investigate the composition and chemical state of the surface of the synthesized samples since it is frequently claimed that the species at the surface play a critical role in oxidation reactions.^31^Figure 5 presents the Co 2p_3/2_, O 1s, and Ru 3p_3/2_ (for the 1Ru/Co_3_O_4_ sample) XPS spectra of the as-prepared catalysts. The Co 2p_3/2_ signal could be deconvoluted into five peaks. The peak at 779.8 eV was related to surface Co^3+^ species in octahedral sites, whereas the signal at 780.8 eV was ascribed to surface Co^2+^ species in tetrahedral sites. The contribution at 781.9 eV was correlated to the presence of CoO species generated by reduction under vacuum conditions in the XPS chamber. For all samples, its abundance was lower than 20% of the total amount of cobalt. Finally, the lower intensity peaks at 783.8 and 789.5 eV were shakeup satellites of CoO and Co_3_O_4_, respectively.^32^ On the other hand, the O 1s spectra were characterized by broad signals, which evidenced the presence of various oxygen species with different chemical states. Thus, the bands at 530.0 and 530.8 eV were ascribed to lattice and adsorbed oxygen species, while the signals at 531.9 and 532.9 eV were correlated to the presence of adsorbed carbonates and water, respectively.^33^ On the Ru 3p_3/2_ spectra, only one signal centered at 463.6 eV could be observed, which was correlated to the Ru^4+^ cation.^12^ No peaks associated with metallic Ru or other oxidation states of ruthenium were noticed, evidencing that the Ru species on the catalyst surface were essentially in the form of RuO_2_. The amount of Ru in the 1Ru/Co_3_O_4_ catalyst was estimated to be about 8 wt % with a surface Ru/Co molar ratio of 0.09, while the bulk Ru/Co molar ratio (calculated from EDX results) was 0.008, evidencing that the ruthenium species were mainly on the catalyst surface, without entering into the inner structure. The Co^2+^/Co^3+^ and O_ads_/O_latt_ molar ratios for the samples are shown in Table 2. As expected, the population of adsorbed oxygen species was controlled by a higher amount of cobalt in low oxidation state (Co^2+^). Moreover, the Co^2+^/Co^3+^ ratio appreciably increased after the addition of Ru, from 0.64 to 0.81. This observation suggested that the Ru species interacted with the Co_3_O_4_ surface causing the reduction of Co^3+^ species to Co^2+^ species. In the same line, the O_ads_/O_latt_ ratio increased from 0.63 for bare Co_3_O_4_ to 0.81 after Ru incorporation. This increase in surface oxygen vacancies (also confirmed by H_2_-TPR and O_2_-TPD) was due to the formation of the Ru–O–Co bond, which caused disorder in the Co_3_O_4_ lattice and favored the creation of structural defects.^12^ Moreover, the electrons flow from Ru atoms to Co atoms through the Ru–O–Co bond provoked a rise in the surface Co^2+^ species. This assumption was also confirmed by the slight shift toward lower binding energy (∼0.1 eV) of the Co 2p_3/2_ signal after the incorporation of Ru.^34,35^

**Table 2:** Surface Composition of the Modified Co_3_O_4_ Catalysts

catalyst	Co^2+^/Co^3+^ molar ratio	O_ads_/O_latt_ molar ratio	Ru^4+^, wt %	Ru/Co molar ratio
Co_3_O_4_	0.64	0.63		
1Ru/Co_3_O_4_	0.81	0.81	8	0.09
Co_3_O_4_–F	0.67	0.66		
0.5Ru/Co_3_O_4_–F	0.75	0.71	6	0.09
0.75Ru/Co_3_O_4_–F	0.70	0.67	9	0.11
1Ru/Co_3_O_4_–F	0.79	0.77	10	0.12

### 3.2 Catalytic Behavior of Co_3_O_4_ and 1Ru/Co_3_O_4_ Catalysts

**Figure 6:**
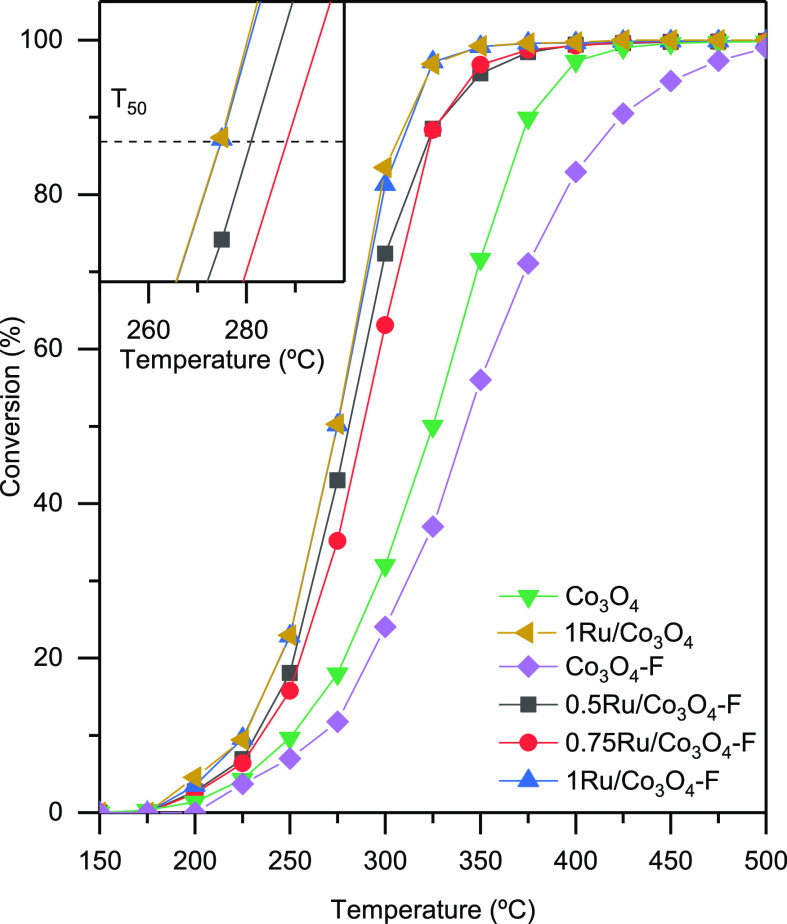
Light-off curves of the modified Co_3_O_4_ catalysts.

The catalytic efficiency for the oxidation of 1,2-DCE was evaluated by following the conversion to CO_2_ with temperature. 1,2-DCE was chosen as a model for the category of double carbon chlorinated VOCs with a H/Cl ratio of >1. This compound typically exhibits an intermediate ease of oxidation between single-chlorinated double carbon olefins such as vinyl chloride^36^ and polychlorinated olefins such as dichloroethylene and trichloroethylene.^37^Figure 6 shows the light-off curves between 150 and 500 °C at 15,000 h^–1^. The corresponding values of *T*_50_ and *T*_90_ (temperatures needed to attain 50 and 90% conversion to CO_2_, respectively) are included in Table 3.

**Table 3:** Kinetic Results of the Oxidation of DCE over the Modified Co_3_O_4_ Catalysts^a^

catalyst	*T*_50_, °C	*T*_90_, °C	(−*r*_A_), mmol g^–1^ h^–1^	*E*_a_, kJ mol^–1^	ln(*k*_0_)^d^
Co_3_O_4_	325	375	0.29^b^	70 ± 1.3	8.3 ± 0.3
1Ru/Co_3_O_4_	275(280)	310(340)	0.36^c^	92 ± 2.4	14.4 ± 0.6
Co_3_O_4_–F	340	420	0.18^b^	66 ± 1.8	7.0 ± 0.4
0.5Ru/Co_3_O_4_–F	280	330	0.28^c^	86 ± 3.8	12.8 ± 0.8
0.75Ru/Co_3_O_4_–F	290	330	0.25^c^	87 ± 0.6	12.7 ± 0.1
1Ru/Co_3_O_4_–F	275	310	0.36^c^	89 ± 1.9	13.8 ± 0.4

a Results in parentheses corresponded to the second reaction cycle.

b Calculated at 275 °C.

c Calculated at 250 °C.

d *k*_0_ is expressed as mol g^–1^ s^–1^ MPa^–1^.

Pure Co_3_O_4_ exhibited a reasonably good performance with *T*_50_ and *T*_90_ values of 325 and 375 °C, respectively. After the addition of ruthenium, the light-off curve shifted to lower temperatures, leading to the complete oxidation of the pollutant at 310 °C. Therefore, it could be deduced that the incorporation of low amounts of ruthenium introduced beneficial changes essentially related to promotion of oxygen mobility or oxygen vacancies of the spinel that eventually would result in an increased oxidation activity. To verify that the 1Ru/Co_3_O_4_ catalyst is not only active but also stable, a consecutive light-off run was carried out (Figure S2, Supporting Information). The temperature to reach the total oxidation of DCE increased by 30 °C, thus pointing out a significant deactivation of the sample after the first reaction cycle. This loss of activity could be due to the volatilization of a certain amount of RuO_2_ species from the surface of the catalyst. In this sense, EDX analysis of the used sample (submitted to two consecutive light-off tests) revealed a slight decrease in the Ru content from 1 to 0.9 wt %. Similarly, XPS results evidenced a much more marked loss of surface Ru (by 30%), from 8 to 5 wt %. Accordingly, the surface Ru/Co molar ratio dropped from 0.09 to 0.05.

As stated previously, the stability of noble metal-modified transition-metal oxide catalysts can be enhanced by strengthening the interaction between the promoter and the bulk oxide. In this sense, acid-etching is a widely employed approach to improve the anchoring of metal particles.^38^ Thus, in an attempt to obtain highly active and stable Ru/Co_3_O_4_ catalysts for the oxidation of chlorinated compounds, the freshly prepared cobalt oxide was treated with an aqueous HF solution (1.2 M) to upgrade metal–support interactions. Then, Ru species were impregnated on the modified oxide. The effect of HF etching on the physical-chemical properties of the Co_3_O_4_ active phase was extensively examined, and the main results will be discussed below.

### 3.3 Structural Changes of Co_3_O_4_ after HF-Etching and Catalytic Behavior

First, the effect of acid treatment on the textural properties of the parent cobalt oxide was studied. Similar to the fresh Co_3_O_4_ sample, the acid-etched counterpart exhibited a type IV isotherm with H1 hysteresis loop, which suggested that the characteristic mesopore structure was maintained after the acid treatment (Figure S1, Supporting Information). However, a significant loss of specific surface area and pore volume was observed (Table 1). On the other hand, the X-ray diffractogram (Figure 1) of the Co_3_O_4_–F sample displayed the same diffraction signals attributable to the spinel structure (ICDD 00-042-1467). Thus, the HF etching did not provoke the structural collapse of the spinel lattice. Nevertheless, an appreciable increase in crystallite size (from 22 to 35 nm) was noticed. This observation was also found by Li et al., who reported a strengthening in the crystallinity of Mn_3_O_4_–Fe_2_O_3_ after treating the mixed oxides with hydrochloric acid.^39^ To gain further insights into the structure of the cobalt oxide, both fresh and etched samples were additionally examined by Raman spectroscopy as well. Hence, the spectra include five Raman bands (A_1g_ + E_g_ + 3F_2g_) in the 100–800 cm^–1^ range (Figure S3, Supporting Information). After the acid modification, an increase in the full width at half-maximum of the A_1g_ vibration mode was observed, in line with the formation of a highly defective structure.^40^

The influence of the acid treatment on the morphology of the nanotubes was evaluated by SEM. From the inspection of Figure 2a,c, it could be deduced that after HF-etching, the hollow morphology was almost completely destroyed leading to the aggregation of nanoparticles of irregular size. However, the walls of the remaining nanotubes were distinctly rough, which could be due to the generation of surface defects during the acid treatment. In view of the textural and structural results, it could be stated that HF induced an increase in the structural disorder of the Co_3_O_4_ phase.

The bulk oxygen mobility of the acid etched-Co_3_O_4_ was also evaluated by means of H_2_-TPR and O_2_-TPD (both bulk and surface) analyses. The results are shown in Table 1 and Figures 3 and 4. Similar to the bare Co_3_O_4_ sample, the acid-etched counterpart showed the characteristic two-step reduction mechanism. The temperatures of the reduction peaks hardly varied with respect to the untreated sample (260 and 335 °C, respectively). As for the O_2_-TPD analysis, comparable results were also found for both Co_3_O_4_ samples. Two overlapping desorption peaks between 120 and 150 °C (surface-adsorbed oxygen species) and a weaker signal above 350 °C ascribed to surface lattice oxygen species were noticed. Regarding the total quantity of oxygen species, both samples evidenced very similar amounts (41 μmol O_2_ g^–1^ over the fresh Co_3_O_4_, and 40 μmol O_2_ g^–1^ over the modified counterpart). These results pointed out that the amount and mobility of bulk oxygen species barely changed after the acid modification. However, it would be of interest to closely examine the effects of acid modification on the surface of the oxide.

To confirm the generation of defects on the surface, XPS analysis of the Co_3_O_4_–F sample was performed. Following the same procedure described earlier for identification and assignment of deconvoluted signals, the corresponding Co 2p_3/2_ and O 1s XPS spectra are displayed in Figure 5. Interestingly, significantly higher surface Co^2+^/Co^3+^ (0.67) and O_ads_/O_latt_ (0.66) molar ratios were obtained in comparison with those found over the untreated oxide (0.64 and 0.63, respectively), thereby confirming the generation of surface defects by the acid treatment in consistency with both Raman and SEM results. XPS analysis was also useful for determining the eventual accumulation of fluorine atoms on the surface. Judging from the absence of the signal at 686 eV of the F 1s spectrum, it was thus confirmed that these species were not present.

The acid-etched cobalt oxide was catalytically evaluated for the oxidation of DCE. The obtained light-off curve as well as the T_50_ and T_90_ values are included in Figure 6 and Table 3, respectively. After acid treatment, the T_50_ value increased by 15 °C compared with the untreated cobalt oxide. This decrease in activity was also observed when comparing the reaction rate results (under differential conditions) given by the untreated sample (0.29 mmol g^–1^ h^–1^) and the HF-etched sample (0.18 mmol g^–1^ h^–1^). This loss of the catalytic efficiency revealed that the generation of surface defects, induced by the acid treatment, was not enough to make up for the observed decrease in surface area and the enlargement of the Co_3_O_4_ crystallite size. In any case, it should be kept in mind that the main target of the acid treatment of the cobalt oxide was not to obtain an active catalyst in the oxidation of chlorinated compounds, but the generation of structural and surface defects that tentatively may enhance ruthenium dispersion and favor metal–support interaction, thus resulting in remarkably active and stable Ru/Co_3_O_4_ catalysts.

Once the highly defective HF-modified Co_3_O_4_ was prepared, ruthenium was deposited following an impregnation method. To study the influence of the amount of Ru, three catalysts with different loadings, namely, 0.5, 0.75, and 1 wt %, were prepared. The *x*Ru/Co_3_O_4_–F catalysts were thoroughly characterized and evaluated in the oxidation of DCE.

### 3.4 Physical-Chemical Characterization of the *x*Ru/Co_3_O_4_–F Catalysts

After the incorporation of the ruthenium as RuO_2_, the resulting catalysts preserved their textural properties. The specific surface area of the solids slightly increased with respect to that of Co_3_O_4_–F, although no noticeable differences were noted with the Ru loading. Type IV isotherms with H1 hysteresis cycles were also observed (Figure S1, Supporting Information), which suggested that the mesoporous structure was mostly retained upon ruthenium impregnation. Results of BET surface area, pore volume, and mean pore diameter (Table 1) were quite similar for all the Ru-impregnated catalysts, namely, a surface area of around 10 m^2^ g^–1^, a pore volume between 0.02 and 0.03 cm^3^ g^–1^, and a mean pore diameter of about 180–200 Å.

The XRD patterns presented in Figure 1 obviously showed the set of diffraction peaks corresponding to the cubic spinel Co_3_O_4_ oxide, at 2θ = 19.2, 31.4, 37.0, 38.7, 45.0, 55.8, 59.5, and 65.4°. In line with the results for the 1Ru/Co_3_O_4_ sample, no diffraction peaks corresponding to metallic ruthenium or ruthenium oxides were detected, owing to the low concentration and high dispersion of the promoter. All the catalysts exhibited similar values of Co_3_O_4_ crystallite size between 36 and 37 nm (Table 1). Complementarily, the samples were characterized by STEM-HAADF. The corresponding particle size distribution histograms are presented in Figure S4, Supporting Information. The average particle size was calculated from the measurement of a set of particles, typically larger than 150 and never below 100. The obtained values ranged between 31 and 34 nm (Table 1), in fairly good agreement with XRD results. As regards the particle size distribution, it should be pointed out that between 75 and 80% of the particles were smaller than 35 nm for all the catalysts.

The SEM images of the *x*Ru/Co_3_O_4_–F catalysts are shown in Figure 2d–f. A heterogeneous morphology could be found for all the samples, as a mixture of nanotubes and irregular clusters formed by the agglomeration of discrete nanoparticles. On the other hand, when comparing the external morphology of the Ru-modified samples with that of the fresh and acid-etched Co_3_O_4_, several differences could be seen. The walls of the 1Ru/Co_3_O_4_ sample (Figure 2b) were quite smooth. However, the 1Ru/Co_3_O_4_–F catalyst (Figure 2f) exhibited rougher walls, which could be an indication of surface defects.

**Figure 7:**
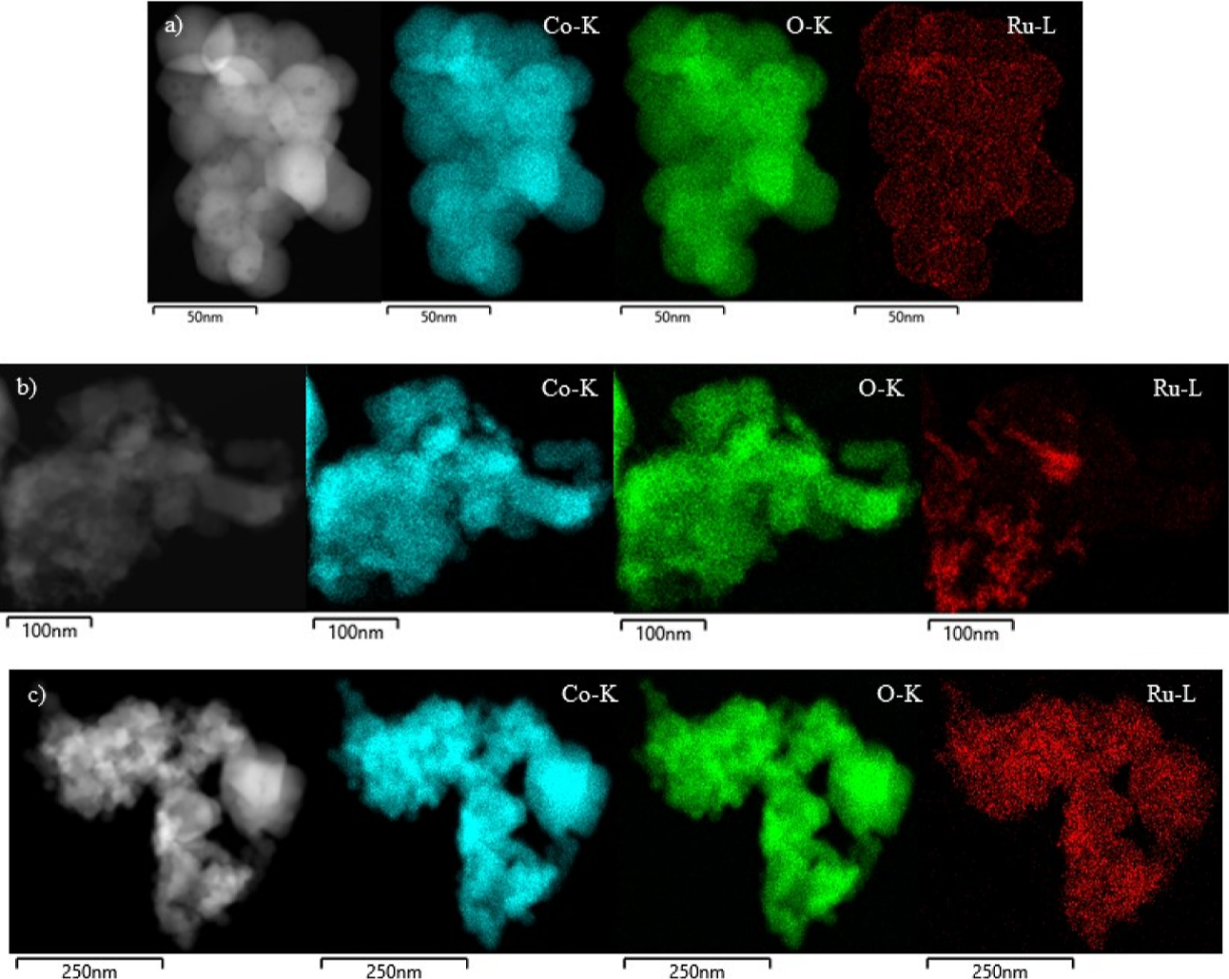
STEM-HAADF images and SEM–EDX maps of the modified Co_3_O_4_ catalysts: (a) 0.5Ru/Co_3_O_4_–F, (b) 0.75Ru/Co_3_O_4_–F, and (c) 1Ru/Co_3_O_4_–F. (Blue) cobalt, (green) oxygen, and (red) ruthenium.

To gain more insights into the dispersion of the Ru species on the surface of the modified cobalt oxide, EDX-mapping images were taken (Figure 7). As far as the 0.5Ru/Co_3_O_4_–F catalyst was concerned, it was observed that ruthenium was homogeneously distributed over the entire surface of the support. As the Ru loading increased (0.75%), this distribution became more heterogeneous since regions with markedly variable Ru density were noted. On the other hand, when increasing the Ru loading up to 1%, a more homogeneous distribution was also observed although with a higher density than that in the 0.5Ru/Co_3_O_4_–F sample. These findings evidenced that the HF etching favored the dispersion of RuO_*x*_ species.

The corresponding H_2_-TPR profiles of the catalysts are displayed in Figure 3. The *x*Ru/Co_3_O_4_–F samples exhibited two well discernible uptakes at 100–180 °C and 180–300 °C assignable to the reduction of Co^3+^ to Co^2+^ and finally to Co^0^. The low-temperature peak was around 140–145 °C, somewhat lower (15 °C) than that over the 1Ru/Co_3_O_4_ catalyst. Besides, a significant distortion in the high-temperature uptake over the acid-etched catalysts was observed. Nevertheless, the total reduction of the *x*Ru/Co_3_O_4_–F and the 1Ru/Co_3_O_4_ samples was fulfilled at the same temperature (300 °C). It was found that, within the experimental error, the observed uptakes (16.8–17.0 mmol H_2_ g^–1^) coincided with the theoretical consumption (16.6 mmol H_2_ g^–1^). Moreover, no obvious differences in the reduction temperatures could be detected as a function of Ru content. According to literature, with promoter loadings above a certain amount, the maxima of the peak reduction temperatures are not affected, and there is no further promotion due to the partial accumulation of ruthenium oxide on the surface of Co_3_O_4_.^41^

Figure 4 includes the O_2_-TPD profiles for the Ru-promoted catalysts. The three samples exhibited a similar desorption profile, with a peak temperature around 550 °C. The amounts of desorbed surface oxygen species were estimated by integrating the areas of the desorption signal below 700 °C (Table 1) and decreased as follows: 1Ru/Co_3_O_4_–F (60 μmol g^–1^) > 0.5Ru/Co_3_O_4_–F (52 μmol g^–1^) > 0.75Ru/Co_3_O_4_–F (49 μmol g^–1^). These results suggested that the oxygen mobility was considerably promoted over the 1Ru/Co_3_O_4_–F sample (very similar to that of the 1Ru/Co_3_O_4_ catalyst, 63 μmol g^–1^) followed by the 0.5Ru/Co_3_O_4_–F and 0.75Ru/Co_3_O_4_–F oxides.

Finally, the surface composition and chemical state of the as-prepared catalysts were analyzed by XPS. The Co 2p_3/2_, O 1s, and Ru 3p_3/2_ XPS spectra are shown in Figure 5. The signals were deconvoluted according to the procedure explained before. It must be highlighted that again only Ru^4+^ species were detected over the various *x*Ru/Co_3_O_4_–F catalysts. The Co^2+^/Co^3+^ and O_ads_/O_latt_ molar ratios as well as the surface Ru^4+^ content over the samples are shown in Table 2. It was found that the 1Ru/Co_3_O_4_–F catalyst exhibited the highest Co^2+^/Co^3+^ and O_ads_/O_latt_ molar ratios on the surface followed by the 0.5Ru/Co_3_O_4_–F sample, which was expected to be beneficial for Cl-VOC oxidation. Conversely, the 0.75Ru/Co_3_O_4_–F catalyst showed the lowest molar ratios. As previously stated, after the incorporation of ruthenium, the amount of oxygen vacancies as well as the population of Co^2+^ species increased owing to the electrons flow from Ru atoms to Co atoms through the Ru–O–Co bond. This assumption was further corroborated by Raman analysis. After the ruthenium addition, the A_1g_ vibration mode of Co–O red-shifted (Figure S5, Supporting Information), which suggested that the RuO_2_ can sway the stretching vibration of Co–O bond, implying the creation of Ru–O–Co bonds.^42^ On the other hand, the amount of surface Ru was higher in the 1Ru/Co_3_O_4_–F sample (10 wt %) than that in the 1Ru/Co_3_O_4_ catalyst (8 wt %), which pointed out that the acid treatment favored the dispersion of the Ru species on the surface of the catalyst.

### 3.5 Catalytic Behavior of the *x*Ru/Co_3_O_4_–F Catalysts

The behavior of the *x*Ru/Co_3_O_4_–F catalysts for DCE oxidation to CO_2_ as a function of temperature is shown in Figure 6. Besides, the corresponding values of *T*_50_ and *T*_90_ (temperatures needed to attain 50 and 90% conversion, respectively) are listed in Table 3. The modified cobalt catalysts showed an excellent performance since in all cases, the chlorinated ethane was fully oxidized to CO_2_ (>90% conversion) below 330 °C. Moreover, after the incorporation of ruthenium oxide, the catalytic efficiency of the samples was appreciably boosted since the *T*_50_ values decreased by 50–65 °C in comparison with the Co_3_O_4_–F sample.

Judging from the catalytic results presented in Table 3, significant differences in the catalytic performance were noticed as a function of the amount of ruthenium. The temperature required for 50% conversion was in the 275–290 °C range; meanwhile, 90% conversion was achieved between 310 and 330 °C. By considering the *T*_50_ value, the catalytic efficiency of the Ru-promoted cobalt oxide catalysts ranked as follows: 1Ru/Co_3_O_4_–F > 0.5Ru/Co_3_O_4_–F > 0.75Ru/Co_3_O_4_–F. On the other hand, the used samples were characterized by EDX and TPO to evaluate the chlorination of the catalysts as well as the formation of carbonaceous deposits. EDX analysis revealed the presence of small amounts of chlorine (0.15–0.20 wt %) on the samples. On the other hand, TPO measurements coupled to mass spectrometry revealed that the coke generation during the reaction was negligible. Thus, when increasing the temperature with a heating ramp of 10 °C min^–1^ under a controlled atmosphere (5%O_2_/He) up to 800 °C, a mass loss associated with water desorption (*m*/*z* = 18) was detected at around 100 °C. However, at higher temperatures, no noticeable mass change was observed. Accordingly, the profile of the CO_2_ signal (*m*/*z* = 44), which could be formed because of the combustion of the eventually formed carbonaceous deposits, in the corresponding mass spectrum was flat. These findings were also consistent with the C balance, which was around 100% (within the experimental error) throughout the whole reaction run.

**Figure 8:**
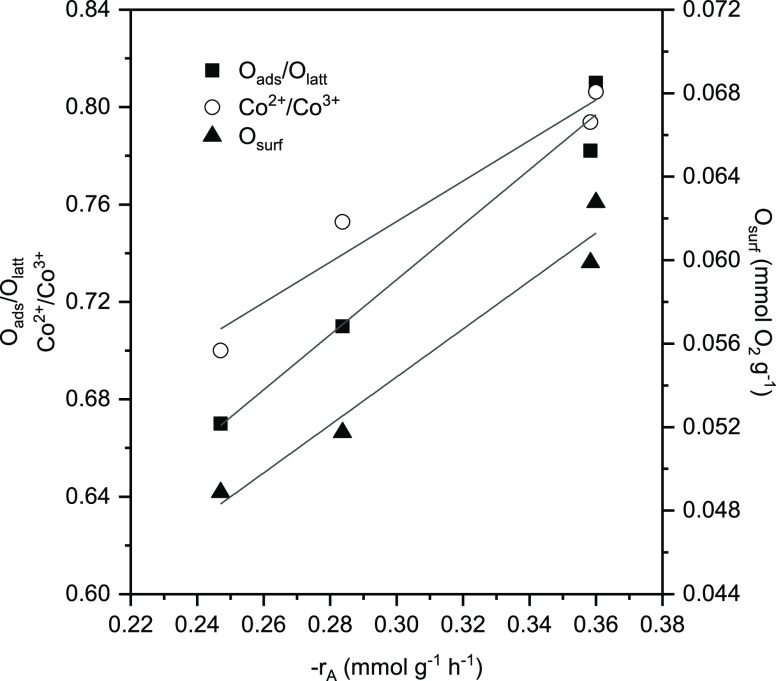
Relationship among the specific reaction rate, the amount of active oxygen species, and the Co^2+^/Co^3+^ and O_ads_/O_latt_ molar ratios.

The intrinsic activity of the samples was better assessed in terms of the measured reaction rate under differential conditions at 250 °C (when conversion was below 20%). The results presented in Table 3 exhibit the same trend as that dictated by the *T*_50_ values. The 1Ru/Co_3_O_4_–F sample showed the highest reaction rate (0.36 mmol_DCE_ g^–1^ h^–1^) followed by the 0.5Ru/Co_3_O_4_–F and 0.75Ru/Co_3_O_4_–F samples (0.28 and 0.25 mmol_DCE_ g^–1^ h^–1^, respectively). Figure 8 reveals a reasonable correlation among the intrinsic reaction rate, the amount of surface active oxygen species (as estimated by O_2_-TPD analysis and by XPS), and the surface Co^2+^/Co^3+^ molar ratio (as determined by XPS). The results corresponding to the 1Ru/Co_3_O_4_ catalyst were also included. As previously stated, the incorporation of Ru species onto the surface of the Co_3_O_4_ provoked the reduction of Co^3+^ species increasing the amount of surface Co^2+^, which in turn was related to oxygen defects close to the surface. Hence, the promotion of oxygen vacancies can activate gas-phase oxygen molecules to generate active oxygen species.

These correlations between activity and physicochemical properties were better rationalized when the mechanism of catalytic oxidation reactions of chlorinated VOCs over the cobalt catalyst was taken into account. According to literature, first, chlorinated molecules adsorb on Co^2+^ sites. Then, the adsorbed molecule is attacked by the active oxygen species on the surface of the catalyst, which are responsible for breaking the C–Cl bond. Thus, the Cl species fill the oxygen vacancy on the catalyst surface. Finally, Ru species facilitate the release of Cl species via the Deacon reaction, and active oxygen species on the catalyst surface play a crucial role in fully converting adsorbed hydrocarbon species into CO_2_.^15^ As demonstrated previously, the population of Co^2+^ and O_ads_ species increased after Ru incorporation, which was favorable for DCE oxidation since the surface Co^2+^ sites were the active sites for DCE adsorption, and O_ads_ species were the responsible for the oxidation of the chlorinated VOC to CO_2_.

**Figure 9:**
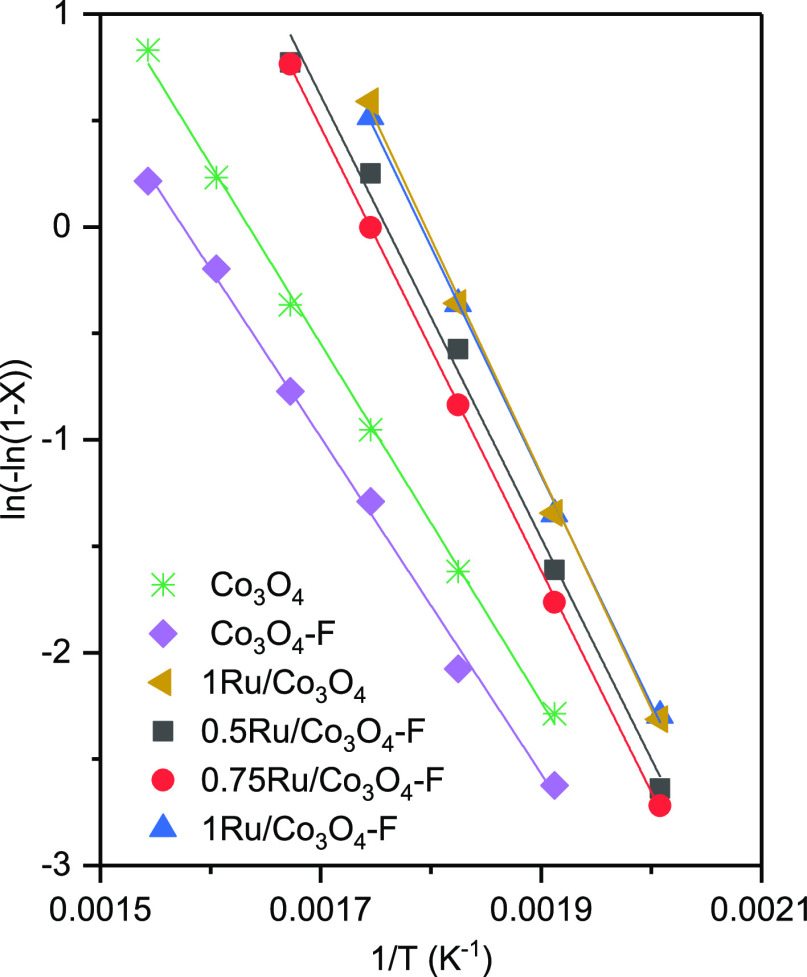
Pseudo-first-order fit for the experimental data obtained over the modified Co_3_O_4_ catalysts.

Kinetic studies were accomplished considering pseudo-first-order kinetics in DCE, zero order for oxygen (owing to its large concentration), and an Arrhenius dependence of the rate constant. These kinetic models are frequently reported in the literature for Cl-VOC oxidation and used for catalyst comparison and preliminary reactor design.^9,43^ These conditions were assumed to be valid for the catalytic oxidation of DCE in chlorinated VOC-air diluted mixtures over the examined Ru-promoted cobalt catalysts. To determine the kinetic parameters (i.e., activation energy and pre-exponential factor), the integral method (conversion between 5 and 80%) was applied. Consequently, if the change in density of the reactant gas due to reaction is considered negligible (since DCE is very diluted), then the following linearized equation can be derived for the integral reactor
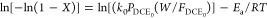
1where *X* is the fractional conversion of DCE, *k*_0_ is the pre-exponential factor of the Arrhenius equation, and *W*/*F*_DCE,0_ is the w8 hly space velocity. Pre-exponential factor and apparent activation energies estimated from eq 1 are included in Table 3. In Figure 9, the goodness of the numerical fit for the kinetic study is shown. The obtained values for the activation energy were in the 86–92 kJ mol^–1^ range for the Ru-promoted catalysts and about 70 kJ mol^–1^ for the parent cobalt oxides, quite similar for all the samples, which evidenced that the reaction mechanism was the same irrespective of the investigated catalyst. These *E*_a_ values were comparable with those reported in the literature for the combustion of chlorinated VOCs over Co_3_O_4_ and Ru-promoted Co_3_O_4_ catalysts^6,44−47^ (Table 4). Since all the catalysts exhibited a relatively comparable value of the apparent activation energy, the ratio between the values of the pre-exponential factor was taken as an indication of the relative activity of the studied oxides. Thus, it was observed that 1Ru/Co_3_O_4_–F was about three times more active than 0.75Ru/Co_3_O_4_–F and about 2.7 times more active than 0.5Ru/Co_3_O_4_–F. These results corroborate the trend derived from the light-off curves and the specific reaction rate. On the other hand, theoretical conversion values could be estimated from the kinetic model and compared with those obtained experimentally. Figure S6, Supporting Information shows a good relationship between the experimental and theoretical conversion values, which validated the proposed kinetic rate expression over the studied temperature range.

**Table 4:** Comparison of the Activation Energies in Chlorinated VOC Oxidation with Cobalt Oxide Catalysts Reported in the Literature

catalyst	chlorinated VOC	Cl-VOC concentration, ppm	WHSV, mL g^–1^ h^–1^	*E*_a_, kJ mol^–1^	reference
*x*Ru/Co_3_O_4_–F	DCE	1000	35,000	86–89	this work
Co_3_O_4_-7P	DCM	3000	60,000	107	(6)
ZIF-Co	VC	1000	15,000	101	(44)
Co_3_O_4_	CB	1000	60,000	71	(45)
Co_3_O_4_	VC	1000	15,000	76–84	(46)
RuO_*x*_/Co_3_O_4_–IM	DCE	1000	60,000	96	(47)

The catalysts examined in this study introduce research path on catalytic systems that could be used in oxidation reactions. Although a large number of studies on Ru–Co_3_O_4_ systems for the oxidation of volatile organic compounds with varying chemical nature (both chlorinated and nonchlorinated hydrocarbons) can be found in the literature, very few of them deal with the acid modification of the cobalt spinel to improve the activity and stability of the resultant catalysts. Instead, most of these investigations focus on the variation of the noble metal loading (0.5–1% wt), studying various routes of cobalt oxide synthesis (impregnation, precipitation, and sol–gel), or the use of supports with high-surface area (ZIF-67, titania, or alumina nanosheets).^11,12,15,29,48−50^ This is why we consider that our results can be a very interesting starting point in the development of new strategies for obtaining efficient cobalt catalysts for the oxidation of recalcitrant pollutants. A comparative analysis of similar catalytic systems found in the literature is shown in Table 5. Hence, it was found that Ru–Co_3_O_4_-based catalysts, when operating at relatively high WHSV, are adequate candidates for the removal of light hydrocarbons, aromatic compounds, and chlorocarbons, with oxidation temperatures for full conversion lower than around 300 °C.

**Table 5:** Catalytic Performance of Ru–Co_3_O_4_ Catalysts in the Oxidation of VOCs

catalyst	chlorinated VOC	Cl-VOC concentration, ppm	WHSV, mL g^–1^ h^–1^	*T*_90_, °C	reference
1Ru/Co_3_O_4_–F	DCE	1000	35,000	310	this work
0.5Ru/Co_3_O_4_–F	DCE	1000	35,000	330	this work
1Ru/Co_3_O_4_	VC	1000	12,000	215	(11)
1Ru/Co_3_O_4_	DCB	1000	30,000	300	(12)
0.5Ru/Co_3_O_4_-10F	VC	20,000	30,000	250	(15)
0.5Ru/Co_3_O_4_	DCE	1000	45,000	330	(29)
1Ru-5Co/TiO_2_	benzene	500	60,000	215	(48)
1Ru/Co_3_O_4_-MOF	toluene	1000	60,000	240	(49)
1Ru/CoANS	propane	1000	15,000	260 (T_50_)	(50)

**Figure 10:**
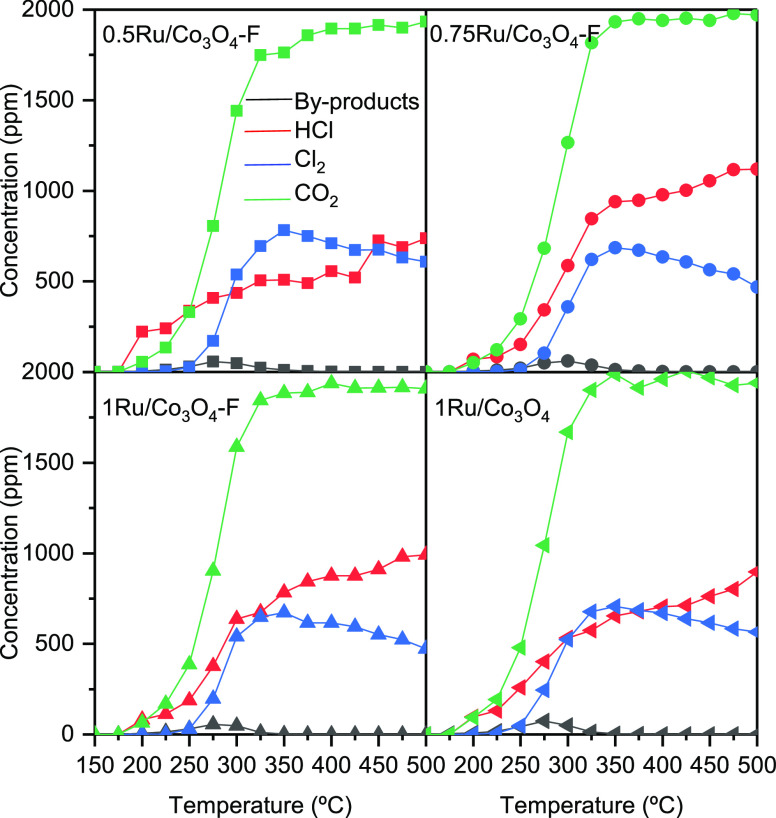
Product distribution during the catalytic oxidation of DCE over the modified Co_3_O_4_ catalysts.

**Figure 11:**
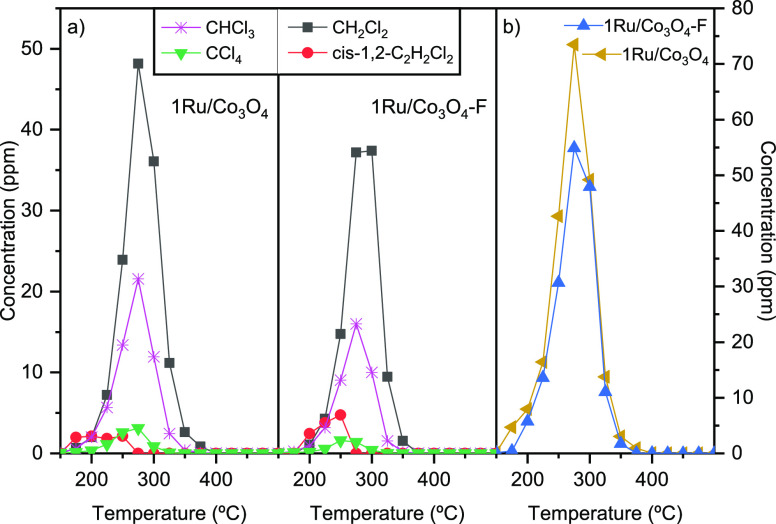
Comparison of the byproducts generated over the 1Ru/Co_3_O_4_ and 1Ru/Co_3_O_4_–F catalysts. (a) Byproducts chemical nature, (b) accumulated byproducts.

The product distribution of the synthesized catalysts as a function of temperature was also examined. For the Ru-free cobalt oxide catalysts, the main chlorinated byproducts detected were chlorinated methanes (dichloromethane, trichloromethane, and tetrachloromethane) and, to a lesser extent, chlorinated ethylenes such vinyl chloride and *cis*-1,2-dichloroethylene. After the addition of ruthenium (Figure 10), these chlorinated hydrocarbons were also detected [dichloromethane (35–50 ppm), trichloromethane (15–20 ppm), and *cis*-1,2-dichloroethylene and tetrachloromethane (<5 ppm)]. However, their concentration was dramatically decreased. Interestingly, vinyl chloride, which is a known byproduct formed by dehydrochlorination of the feed, was not detected in any case, possibly due to the strong redox ability of the modified catalysts.^51^ Besides, when comparing the product distribution over the two studied 1% Ru supported catalysts (1Ru/Co_3_O_4_ and 1Ru/Co_3_O_4_–F) (Figure 11), it was observed that the generation of byproducts was minimized over the whole temperature range studied when the HF-etched Co_3_O_4_ was used. These results highlighted the beneficial role of acid treatment in product selectivity. As aforementioned, HF-treatment improved the dispersion and increased the amount of Ru species on the Co_3_O_4_ surface. These Ru ions were responsible for removing the Cl species from the catalyst surface through the Deacon reaction. Thus, a higher concentration of Ru species could activate the desorption of Cl species from the catalyst surface and lead to a lower polychlorinated byproduct generation. On the other hand, the main chlorinated deep oxidation products were Cl_2_ and HCl. Under complete DCE conversion conditions (325 °C), the observed Cl_2_/HCl ratio was in the 1–1.4 range, except for the 0.75Ru/Co_3_O_4_–F sample, which exhibited a significantly lower value (0.7). These values were appreciably higher than that obtained for bare Co_3_O_4_ (0.7), attributable to the high activity of ruthenium oxide in the Deacon reaction.^13^ It is worth highlighting that no CO was detected over the whole temperature range, and CO_2_ was the main nonchlorinated oxidation product.

**Figure 12:**
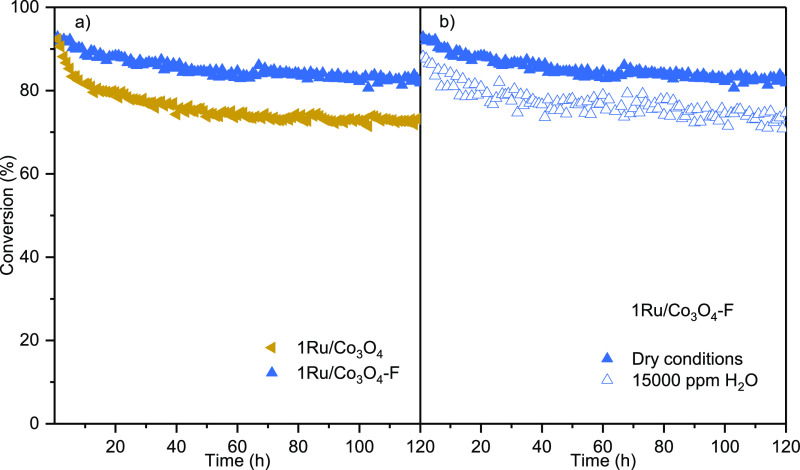
Stability in DCE oxidation at 300 °C for 120 h. (a) Comparison between 1Ru/Co_3_O_4_ and 1Ru/Co_3_O_4_–F samples, (b) 1Ru/Co_3_O_4_–F under dry and humid conditions.

Finally, the catalytic stability of the most efficient promoted-oxide, namely, the 1Ru/Co_3_O_4_–F catalyst, was investigated by analyzing the evolution of conversion to CO_2_ with time on stream at 300 °C for 120 h (Figure 12). This run evidenced that conversion slightly decreased (about 10%) during the first 40 h. However, during the remaining 80 h, it remained stable (83%). The used 1Ru/Co_3_O_4_–F sample was characterized by XRD, EDX, N_2_ physisorption, and XPS. The crystallite size slightly enlarged from 36 nm on the fresh catalyst to 37 nm on the used sample. Interestingly, no diffraction peaks corresponding to the formation of less active species such as CoO or RuCl_2_ were observed. In terms of textural properties, the stability test did not affect the surface area, which remained unaltered at 10 m^2^ g^–1^. Finally, in view of the results shown in Figure 12, it could be expected that this relatively long stability test did not provoke a massive volatilization of the RuO_2_ species. However, to confirm this assumption, EDX analysis was carried out on the used sample. The Ru content hardly changed after the 120 h test (both fresh and used samples showed a ruthenium content of about 0.9 wt %). Consequently, it was reasonable to conclude that the surface disorder induced by the acid-etching of the cobalt oxide improved the anchorage of Ru particles on the oxide and ultimately the stability of the catalyst. Likewise, the presence of small amounts of chlorine (0.25 wt %) was evidenced. However, it must be pointed out that the concentration of chlorine on the surface of the catalyst was noticeably higher (5.4 wt %) than that in the bulk. This accumulation of chlorine was probably responsible for the slight loss of activity during the first hours of the stability test. In fact, when the chlorine concentration in the sample after 20 h of reaction and at the end of the stability test (120 h) was compared, this hardly increased (4.5 and 5.4 wt %, respectively). In addition, similar results were found for the Cl/Ru molar ratio (about 1.9).

The good stability of this sample was best assessed when comparing it with the catalytic performance shown by its untreated counterpart (1Ru/Co_3_O_4_) under the same reaction conditions (Figure 12a). The results revealed that although both samples exhibited the same initial conversion value (∼92%), the 1Ru/Co_3_O_4_ sample underwent a more severe deactivation over time until it stabilized at around 73% conversion. This value was significantly lower than that shown by the 1Ru/Co_3_O_4_–F sample (83%). Therefore, it could be concluded that the acid treatment of the cobalt oxide improved the thermal stability of the Ru species due to a stronger interaction between Co_3_O_4_ and RuO_2_ species, which led to a better stability of the 1Ru/Co_3_O_4_–F catalyst when operating during prolonged reaction time intervals.

The durability of this sample (120 h) was also evaluated at 300 °C under humid conditions (15,000 ppm of H_2_O) (Figure 12b). After a slightly progressive decrease in conversion during the first 40 h, then it remained constant at 75%. This conversion value was somewhat lower than that obtained at the same temperature under dry conditions (83%), possibly due to the competitive adsorption of DCE and H_2_O molecules.^52^ Indeed, the surface chlorination of the catalysts under humid conditions was appreciably lower (2.0 wt %) owing to efficient role of H_2_O in cleaning the catalyst surface. These results, however, indicated that the optimal 1Ru/Co_3_O_4_–F catalyst exhibited both good catalytic stability and H_2_O resistance in the catalytic oxidation of DCE at low temperatures. As for Cl_2_/HCl selectivity, the formation of hydrogen chloride at the expense of molecular chlorine was substantially favored in the presence of excess water as the Cl_2_/HCl molar ratio decreased from 0.8 under dry conditions to 0.5 under humid conditions. Moreover, the byproduct generation was dramatically diminished after the addition of water. Only 30 ppm of dichloromethane was detected after the addition of water; meanwhile, its concentration was about 110 ppm under dry conditions. Besides, the formation of trichloromethane was reduced from 30 to 10 ppm under humid conditions, and the generation tetrachloromethane was totally suppressed. This reduction in the byproduct yields could be explained by the inhibition of the reactivity of Cl_2_ by water molecules as a hydrogen source.^53^

## 4 Conclusions

A series of Ru-promoted (with different Ru loadings) cobalt oxide catalysts with controlled nanomorphology were synthesized by a precipitation route based on the Kirkendall effect followed by an acid treatment (HF) and an optimized impregnation method. The prepared samples were exhaustively characterized (N_2_ physisorption, XRD, SEM, STEM-HAADF, EDX, H_2_-TPR, O_2_-TPD, XPS, and TPO) and evaluated for total oxidation of DCE at 15,000 h^–1^. All the modified-oxides exhibited a notable activity, achieving the complete conversion of the pollutant to deep oxidation products at temperatures lower than 330 °C. Ruthenium incorporation enhanced oxygen mobility, both at bulk and surface level, and increased the population of Co^2+^ and O_ads_ species at the surface which upgraded the oxidation activity owing to the large population of available active surface oxygen species. Furthermore, the acid treatment considerably improved the dispersion of the ruthenium by preferentially depositing it on the outermost surface and strengthened the interaction between the noble metal and the cobalt oxide, leading to a remarkable stability (compared with the untreated counterpart) during a relatively prolonged time on stream (120 h) under dry and humid conditions.

## Supporting Information Available

N_2_ isotherms and pore size distribution of the modified Co_3_O_4_ catalysts; consecutive light-off runs of the 1Ru/Co_3_O_4_ catalyst; Raman spectra of the acid-etched and fresh Co_3_O_4_ catalysts; particle size distribution of the modified Co_3_O_4_ catalysts; Raman spectra of the modified Co_3_O_4_ catalysts; and comparison of experimental data and estimated values from conversions of the rate equation at different temperatures (PDF)

## Abbreviations

DCE: 1,2-dichloroethane
DCM: dichloromethane
VC: vinyl chloride
CB: chlorobenzene
DCB: 1,2-dichlorobenzene
