# Supplementary material for: Promotion of Cobalt Oxide Catalysts by Acid-Etching and Ruthenium Incorporation for Chlorinated VOC Oxidation
Source: Ind Eng Chem Res. 2024 Feb 13;63(7):3003–17. doi: 10.1021/acs.iecr.3c04045 (PMC10885781; doi:10.1021/acs.iecr.3c04045)
Supplement: Supplementary file 1 — ie3c04045_si_001.pdf [file ie3c04045_si_001.pdf]

PROMOTION OF COBALT OXIDE CATALYSTS BY ACID-ETCHING AND RUTHENIUM INCORPORATION FOR CHLORINATED VOC OXIDATION

Amaya Gil-Barbarin, José Ignacio Gutiérrez-Ortiz, Rubén López-Fonseca, Beatriz de Rivas*

Chemical Technologies for Environmental Sustainability Group, Department of Chemical Engineering, Faculty of Science and Technology, University of The Basque Country UPV/EHU, Barrio Sarriena s/n, Leioa, E-48940, Bizkaia, Spain.

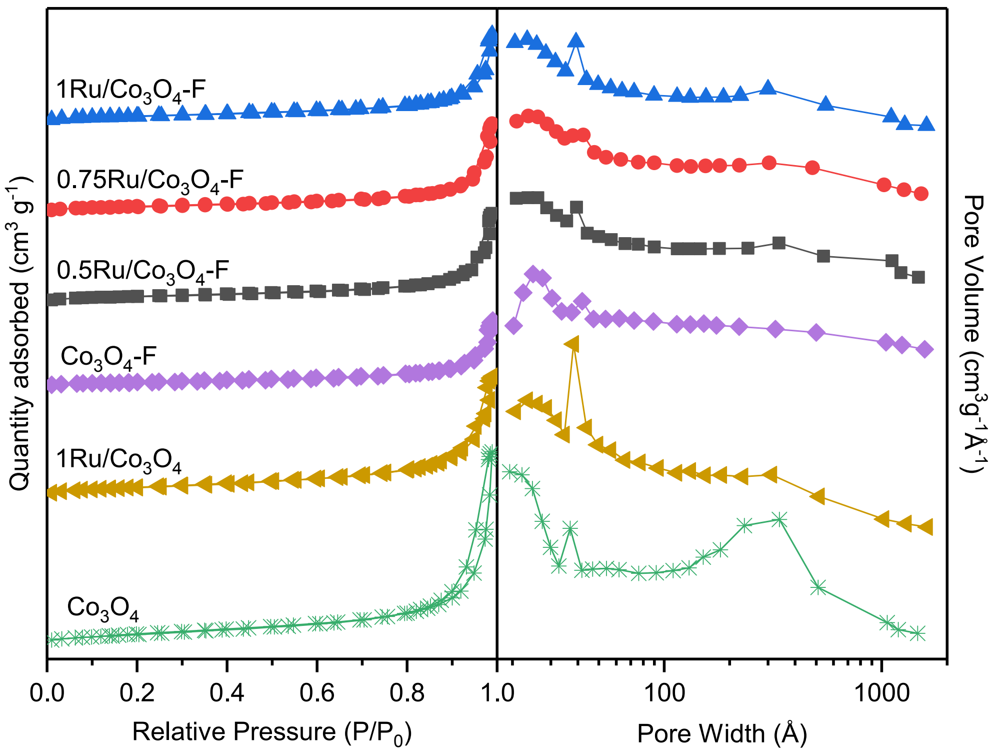

Figure S1. N2 isotherms and pore size distribution of the modified Co_3O_4 catalysts.

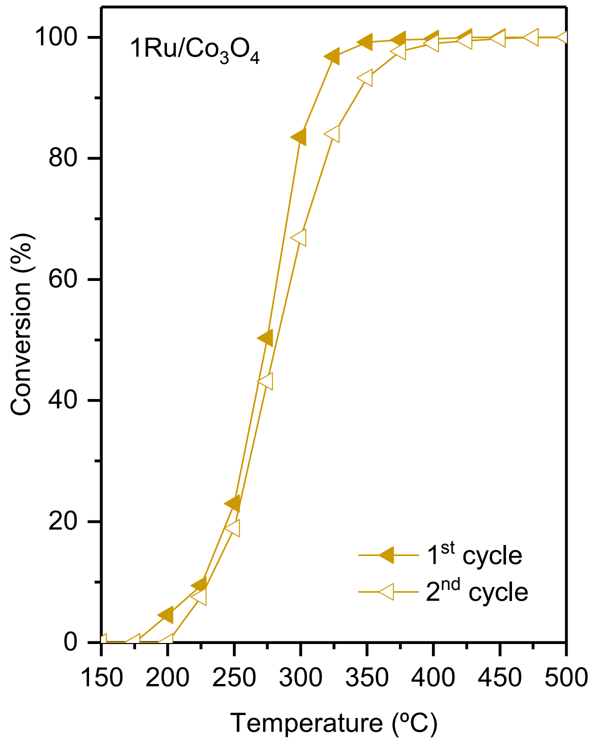

Figure S2. Consecutive light-off runs of the $1Ru/Co_3O_4$ catalyst.

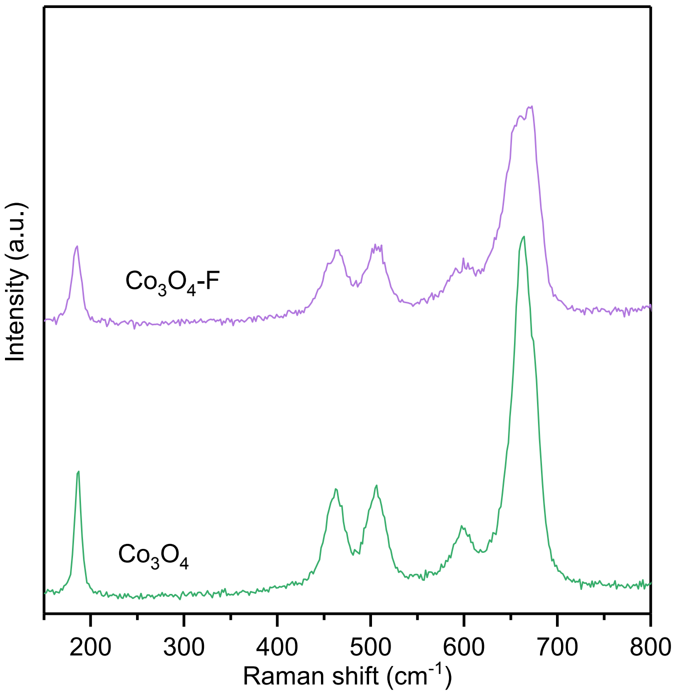

Figure S3. Raman spectra of the acid-etched and fresh Co_3O_4 catalysts.

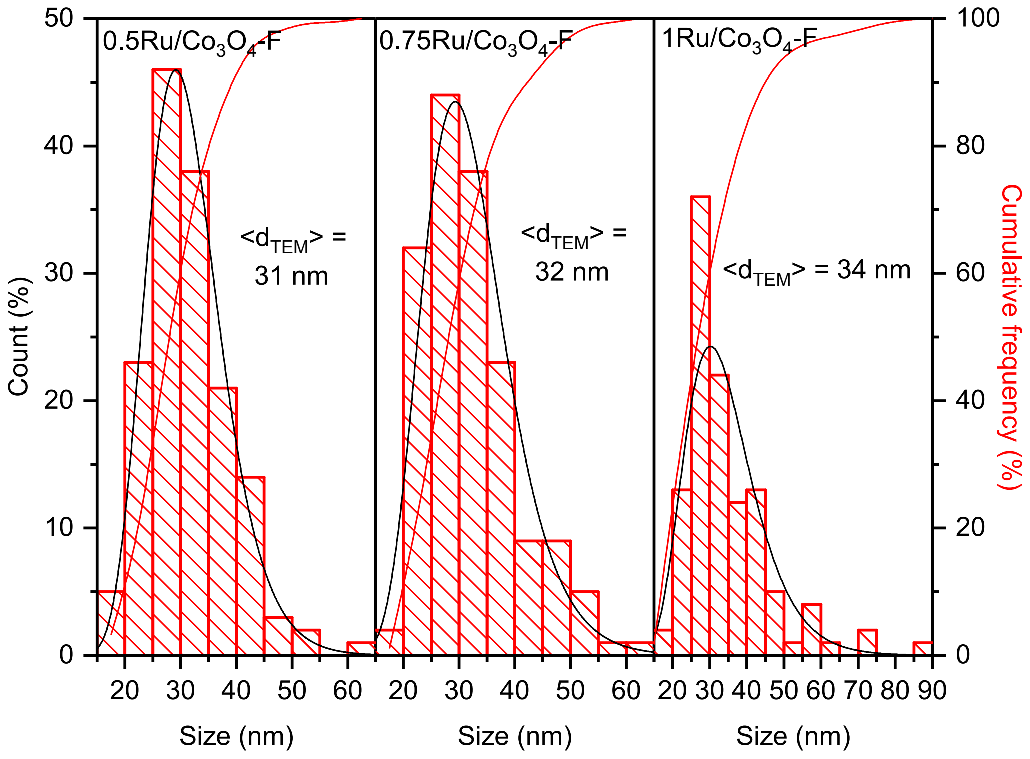

Figure S4. Particle size distribution of the modified Co_3O_4 catalysts.

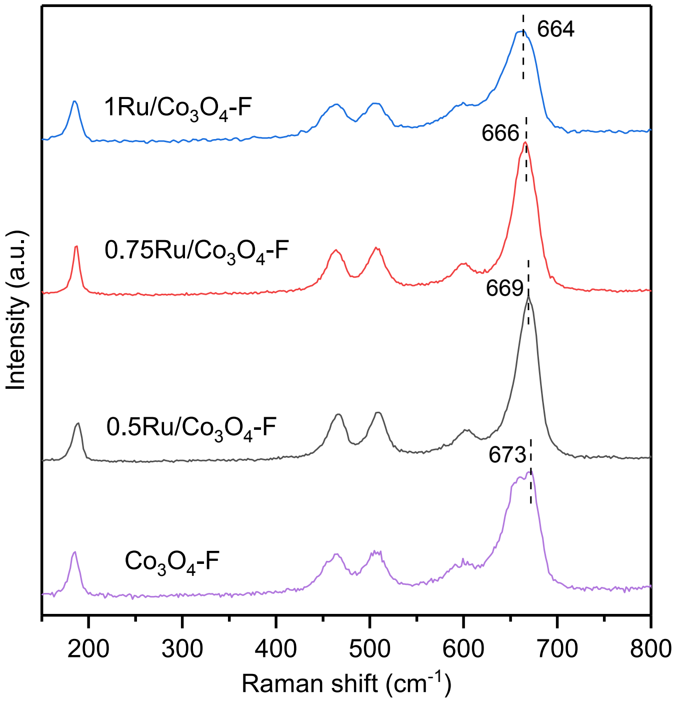

Figure S5. Raman spectra of the modified Co_3O_4 catalysts.

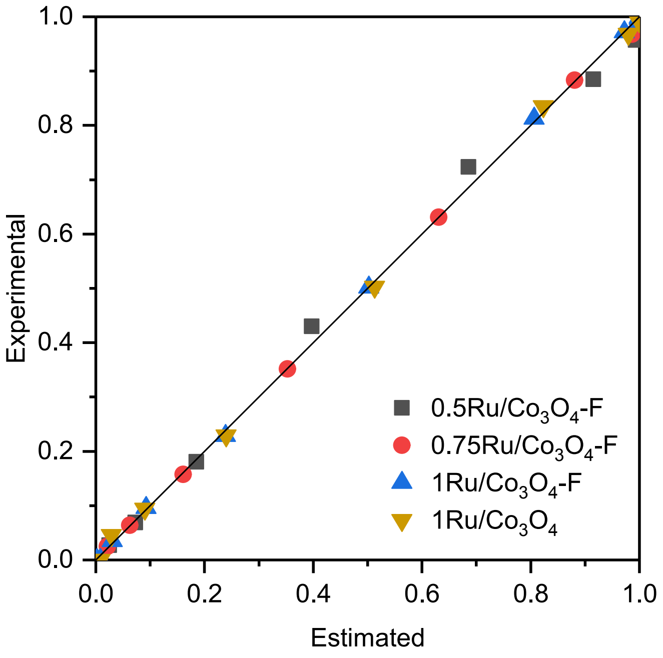

Figure S6. Comparison of experimental data (symbols) and estimated values (solid line; power law model) from conversions of the rate equation at different temperatures.
